# Metal and Metal Halogenide-Filled Single-Walled Carbon Nanotubes: Kinetics, Electronic Properties, Engineering the Fermi Level

**DOI:** 10.3390/nano13010180

**Published:** 2022-12-30

**Authors:** Marianna V. Kharlamova, Christian Kramberger

**Affiliations:** 1 Centre for Advanced Materials Application (CEMEA), Slovak Academy of Sciences, Dúbravská cesta 5807/9, 845 11 Bratislava, Slovakia; 2 Faculty of Physics, University of Vienna, Boltzmanngasse 5, 1090 Vienna, Austria

**Keywords:** metal, metal halogenide, carbon nanotube

## Abstract

Here, we present a review of the major achievements in kinetics, electronic properties, and engineering in the Fermi level of single-walled carbon nanotubes (SWCNTs). Firstly, the kinetics of metal-filled SWCNTs were revealed with precision over several minutes. Secondly, the growth rates of nanotubes were calculated. Thirdly, the activation energies of nanotubes were measured. Fourthly, the methods of the quantitative analysis of the doping level were developed. Indeed, only qualitative analysis has been previously performed. The quantitative analysis allowed us to obtain quantitative data on charge transfer. Fifthly, the correlation between the physical properties, chemical properties, electronic properties of SWCNTs was elucidated.

## 1. Introduction

The filling of single-walled carbon nanotubes (SWCNTs) [1,2,3,4,5,6,7,8,9,10] is made using a gas phase and liquid phase approach. Metals are introduced inside SWCNTs via a solution method, and metal halogenides are filled inside SWCNTs through the melt method [10]. These methods are very promising and allow high filling ratios to be obtained [10]. 

Significant progress has been made in the understanding of kinetics and the electronic properties of filled SWCNTs and the precise control of the Fermi level, which put SWCNTs a step forward toward their use in applications. The kinetics and electronic properties were analyzed using Raman spectroscopy, near edge X-ray absorption fine structure spectroscopy (NEXAFS), X-ray photoelectron spectroscopy (XPS), ultraviolet photoelectron spectroscopy (UPS), and optical absorption spectroscopy (OAS) [11,12,13,14,15,16,17,18,19,20,21,22,23,24,25,26,27,28,29,30,31,32,33,34,35,36,37,38,39,40,41,42,43,44,45,46,47,48,49,50,51,52,53,54,55,56,57,58,59,60,61,62,63,64,65,66,67,68,69,70,71,72,73,74,75,76,77,78,79,80,81,82,83,84,85,86,87,88,89,90,91,92]. It is high time to review tremendous progress that has been made over the last five years [10]. There are five major achievements: Firstly, the kinetics of metal-filled SWCNTs were revealed with the precision of several minutes. Secondly, the growth rates of nanotubes were calculated. Thirdly, the activation energies of nanotubes were measured. Fourthly, the methods of the quantitative analysis of the doping level were developed. Indeed, only qualitative analysis has previously been performed. The quantitative analysis allowed for the obtaining of quantitative data on charge transfer. Fifthly, the correlation between the physical properties, chemical properties, and electronic properties of SWCNTs were elucidated.

The goal of this review is to be the central reference for researchers aiming at a precise control of kinetics and electronic properties toward applications and to stimulate emerging applications utilizing the physical and chemical properties. 

Kinetics and the electronic properties of filled SWCNTs and the engineering of their Fermi level are tailored for their use as applications. This review begins from the overview of metal and metal halogenide filled inside SWCNTs. Then, the kinetics of metal-filled SWCNT growth are discussed. After that, the review discusses the results of the investigation of doping and hybridization effects in SWCNTs filled with metals and metal halogenides using Raman spectroscopy, near edge X-ray absorption fine structure spectroscopy, photoemission spectroscopy, and optical absorption spectroscopy. The methods of the quantification of charge transfer are described, and a discussion of the influence of different metals and metal halogenides on the electronic properties is presented.

## 2. Overview of Substances Filled Inside SWCNTs

To date, more than 150 different substances have been succeeded [93,94,95,96,97,98,99]. The list includes metals and metal halogenides. 

Metals are introduced inside SWCNTs, including alkali metals (potassium [100]), p-metals (bismuth [101]), transition metals (iron [102,102,103,104,105], cobalt [82,104], nickel [106], manganese [104], vanadium [104], molybdenum [104], ruthenium [107], palladium [108], silver [108,109,110,111,112,113,114], copper [109,115], rhenium [116,117,118], gold [108], platinum [108], tungsten [5,104,116,118], osmium [116,118]) and lanthanides (europium [119,120] and erbium [121]). 

Chemical compounds are the largest group of substances introduced inside SWCNTs. The most popular among them are metal halogenides. SWCNTs were filled with metal fluorides (SnF_2_ [122]), metal chlorides (sodium/cesium/copper/silver/tantal)Cl [113,123,124,125,126,127,128,129,130,131,132,133], (manganese/iron/cobalt/nickel/zinc/cadmium/mercury/palladium/lead)Cl_2_ [108,126,127,134,135,136,137,138,139,140,141,142,143,144,145], (iron/yttrium/ruthenium/gold/lanthan/neodym/samarium/europium/gadolinium/terbium/thulium/praseodymium/holmium/erbium/ytterbium/luthetium)Cl_3_, Al_2_Cl_6_ [102,103,107,108,121,126,127,134,139,142,146,147,148,149,150,151,152], (zirkonium/hafnium/platinum/thorium)Cl_4_ [108,127,139,153,154,155,156], MoCl_5_ [126], WCl_6_ [126,127], (KCl)_x_(UCl_4_)_y_ [113,157], metal bromides (cesium/copper/silver)Br [113,123,124,127], (manganese/iron/cobalt/nickel/zinc/cadmium/lead)Br_2_ [135,136,137,138,141,143,145,158,159]), TbBr_3_ [151], LuBr_3_ [152], metal iodides (lithium/sodium/potassium/rubidium/cesium/copper/silver)I [123,124,127,154,155,156,157,160,161,162,163,164,165,166,167,168], (calcium/strontium/barium/iron/cobalt/zinc/cadmium/lead)I_2_ [137,138,141,145,154,155,156,169,170,171], (lanthan/terbium/luthetium)I_3_, Al_2_I_6_ [139,151,152,172,173], SnI_4_ [126,127]), their mixtures [113,126,127,154,157,160,174,175], and other substances [176,177,178,179,180]. 

## 3. Kinetics

**Figure 1:**
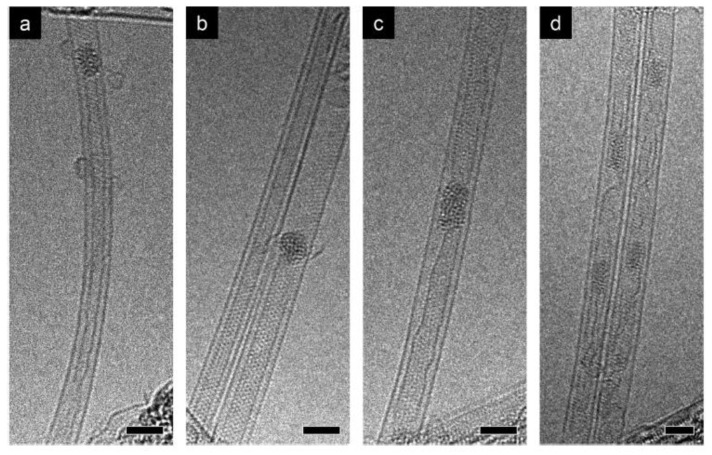
Transmission electron microscopy of cobalt clusters annealed at 550 °C for 2 h: (**a,b**) an inner tube; (**c,d**) metal clusters with graphitic structures. Scale bars, 2 nm. Reprinted from Kharlamova, M.V., et al. Chiral vector and metal catalyst-dependent growth kinetics of single-walled carbon nanotube, Carbon. 2018. V. 133. P. 283-292, Copyright (2018), with permission from Elsevier [98].

The kinetics of the growth of carbon nanotubes inside (C_5_H_5_)_2_Ni- and (C_5_H_5_)_2_Co-filled SWCNTs were investigated [98,99]. The growth rates were calculated, and the activation energies on nickel and cobalt clusters were measured to be between 0.5 and 2.7 eV. It was shown that metal clusters catalyze the growth of nanotubes that continue for long hours. Metal clusters grow in size with time and increasing annealing temperature. The investigations were performed at temperature between 480 and 640 °C for nickel- and cobalt-filled SWCNTs. The duration of growth was between 2 min and 5 days; however, it can continue for an even longer period of investigation. Figure 1 shows the initial metal clusters inside carbon nanotubes. They are seen as the dark contrast elements in the images.

## 4. Electronic Properties

### 4.1. Filling of SWCNTs with Metals 

The encapsulation of metals inside SWCNTs was performed in a saturated solution of metal nitrate (AgNO_3_ [109,110,111,112,179] or Cu(NO_3_)_2_ [109,115]). 

### 4.2. Filling of SWCNTs with Metal Halogenides

The embedding of metal halogenides inside SWCNTs was performed using the melt method. Sealed quartz ampoule was heated above the melting temperature (Table 1), kept at this temperature for some time, and then cooled down. The filling of the compound inside SWCNTs occurred via capillary forces. This filling method allowed nanocomposites with a large filling ratio (up to 90%) of SWCNTs to be obtained. The control of the cooling procedure of the ampoule allowed for the crystallization of the salt inside SWCNTs and for one-dimensional nanocrystals to be obtained.

### 4.3. Doping and Hybridization Effects

#### 4.3.1. Optical Absorption Spectroscopy

The optical absorption spectroscopy (OAS) is an informative method used for the investigation of the electronic properties of filled SWCNTs. It provides data about charge transfer in filled SWCNTs. Many samples of filled SWCNTs can be investigated, and the method is simple and quick. A comparison of the spectra of pristine and filled SWCNTs reveals the modifications of the electronic structure of carbon nanotubes. 

The OAS investigated SWCNTs filled with iron halogenides [138], cobalt bromide [158], zinc halogenides [137], silver halogenides [123], cadmium halogenides [141], CuCl [129], copper halogenides [124], PrCl_3_ [150], and TbCl_3_ [142]. In most cases, changes in the spectrum were attributed to the modification of the electronic properties of SWCNTs. 

**Figure 2:**
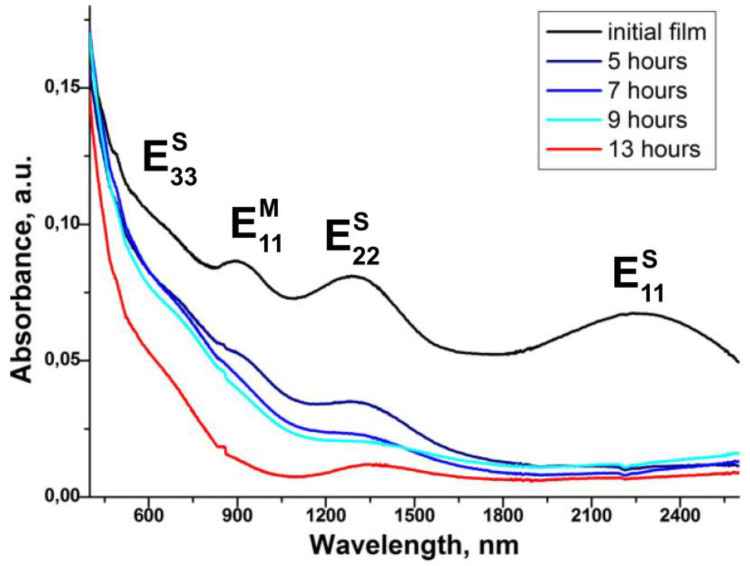
The OAS spectra evolution upon exposure to CuCl gas. Reprinted with permission from [129], copyright 2015 Wiley-VCH Verlag GmbH & Co. KGaA, Weinheim.

Figure 2 compares the OAS spectra of pristine and CuCl-filled SWCNTs with increasing exposure time [129]. There are $E_{11}^{S}$, $E_{22}^{S}$, $E_{33}^{S}$, and $E_{11}^{M}$ absorption bands. The $E_{11}^{S}$ absorption band vanishes after 5 h of exposure to CuCl gas; moreover, with increasing exposure time, other absorption bands are also slightly reduced. This was attributed to canceling optical transitions [129]. 

The suppression of the $E_{11}^{S}$ absorption band was also revealed [123,124,129,135,136,137,138,141,142,158]. Taking into account these data, the authors made a conclusion about doping. For metal chalcogenide-filled SWCNTs, there are no noticeable changes [176,179,181,182,183,184,185,186,187,188]. 

#### 4.3.2. Raman Spectroscopy

Raman spectroscopy is a very useful method of investigation for examining the electronic properties of filled SWCNTs. It is a simple, nondestructive, and informative technique used to investigate the vibronic properties of carbon nanotubes upon filling. The nanotubes have a radial breathing mode (RBM) and D, G, 2D bands of Raman spectra, which are characteristic for a nanotube with a certain diameter, metallicity type, and chiral angle. 

**Figure 3:**
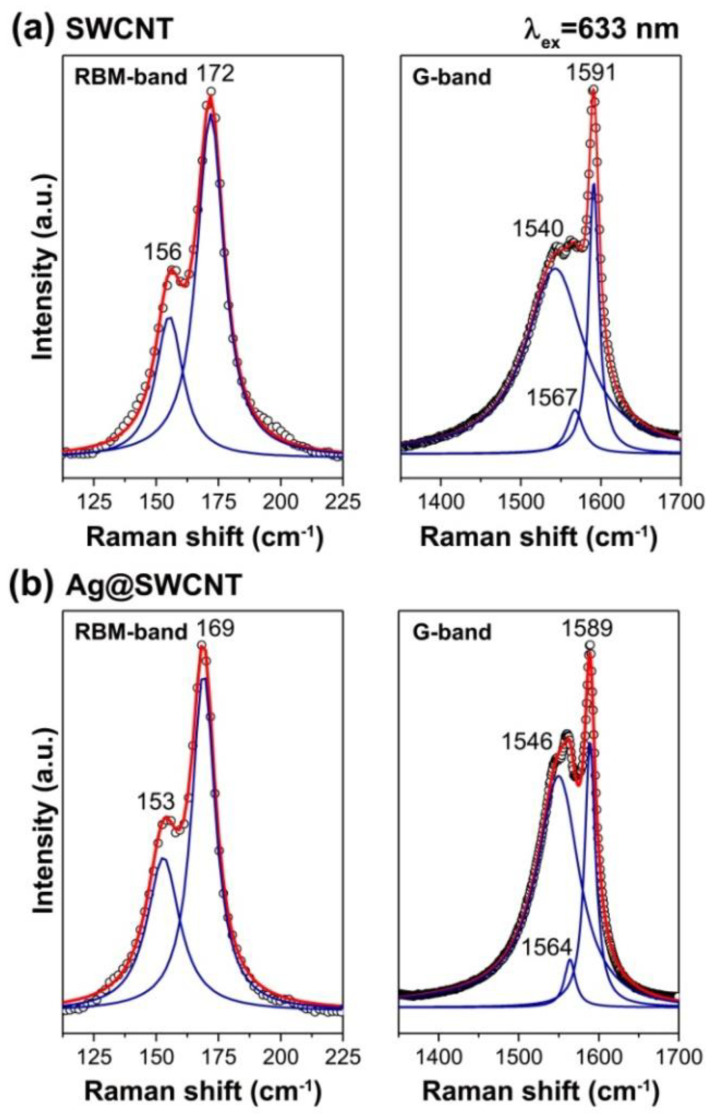
Raman spectra of the pristine SWCNTs (**a**) and silver-filled SWCNTs (**b**) recorded at 1.96 eV (λ_ex_ = 633 nm). The peak positions are indicated. Reproduced from M. V. Kharlamova et al. Donor doping of single-walled carbon nanotubes by filling of channels with silver, Journal of Experimental and Theoretical Physics, V. 115, № 3, p. 485–491, 2012, Springer Nature [110].

The Raman spectrum of pristine SWCNTs shows two dominant peaks in RBM (C1, and C2) [110]. They are located at 156 and 172 cm^−1^ (Figure 3a) and belong to main-diameter semiconducting and metallic SWCNTs, accordingly [189]. The G-line reveals three components at 1540, 1567, and 1591 cm^−1^ (Figure 3a). The positions of the individual peaks are very close to the predicted peak positions [190,191,192]. The G-band further backs up this interpretation, as this shape is reminiscent of metallic SWCNTs [190,193,194].

The Raman spectra of Ag-filled SWCNTs show differences (Figure 3b). In the RBM-band, there are just minuscule downshifts of the peaks by 3 cm^−1^. In the G-band, there are shifts from 2 to 6 cm^−1^. The relative strength of the metallic component increases from 0.70 to 0.79. There is the charge transfer between the SWCNTs and silver with a rigid band shift as the overall metallicity increases while the resonance conditions are preserved.

Besides silver [109,112,179], there are also similarities to copper [109,115]. This is in line with the n-doping of SWCNTs, which is expected from the lower work functions of metals as compared to nanotubes. 

Raman spectroscopy was applied for manganese halogenides [136,143], iron halogenides [138], CoBr_2_ [158], nickel halogenides [135], ZnCl_2_ [142], zinc halogenides [137], silver halogenides [123], CuCl [129], CuI [161,165], copper halogenides [124], CdCl_2_ [142,144], cadmium halogenides [141], lead halogenides [145], SnF_2_ [122], RbI [168], RbAg_4_I_5_ [175], TbCl_3_ [142,149,151], TbBr_3_, TbI_3_ [151], TmCl_3_ [149,179], PrCl_3_ [149,150], luthetium halogenides [152], and HgCl_2_ [140].

**Figure 4:**
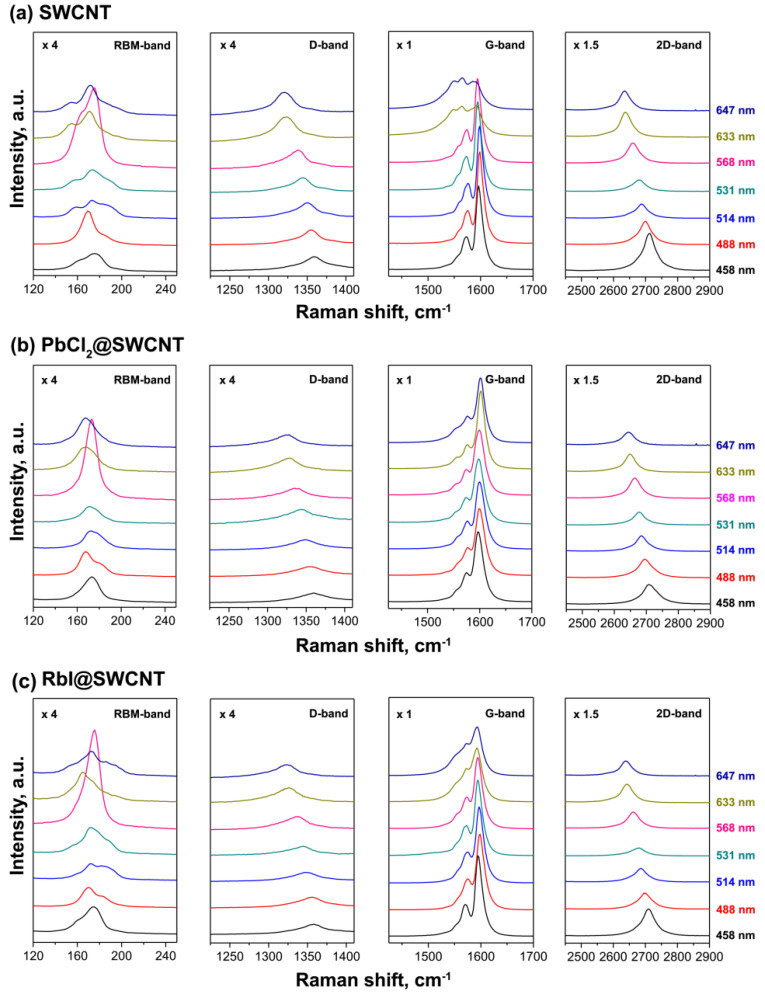
The Raman spectra of the pristine SWCNTs (**a**) [168] (reprinted with permission from [168], copyright 2019 Wiley-VCH Verlag GmbH & Co. KGaA, Weinheim), PbCl_2_@SWCNT (**b**) (reproduced from M. V. Kharlamova et al. Revealing the doping effect of encapsulated lead halogenides on single-walled carbon nanotubes, Appled Physics A, V. 125, article number 320, 2019, Springer Nature [145]) and RbI@SWCNT (**c**) [168] (reprinted with permission from [168], copyright 2019 Wiley-VCH Verlag GmbH & Co. KGaA, Weinheim).

The Raman spectra of pristine SWCNTs, electron acceptor PbCl_2_-filled SWCNTs [145], and electron donor RbI-filled SWCNTs [168] are shown in Figure 4. In the RBM-band, there are shifts in the peaks and an alteration in their relative intensities. In the D and 2D-bands, there are shifts in the peaks and changes in their intensities. The G-band shows changes in the peak positions and modifications inthe peak profiles [145,168].

**Figure 5:**
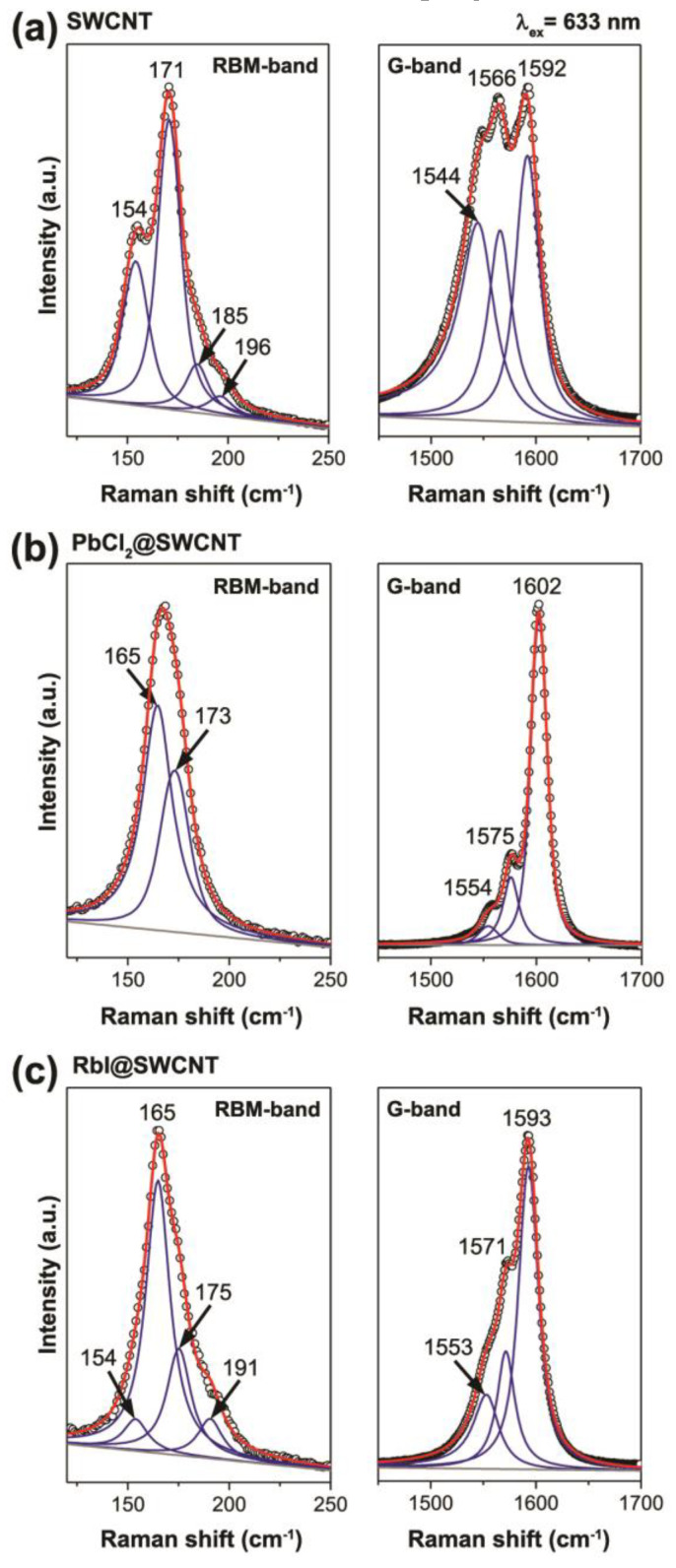
Raman spectra of pristine SWCNTs (**a**) (reproduced from M. V. Kharlamova et al. Revealing the doping effect of encapsulated lead halogenides on single-walled carbon nanotubes, Appled Physics A, V. 125, article number 320, 2019, Springer Nature [145]) and the PbCl_2_-filled SWCNTs (**b**) (reproduced from M. V. Kharlamova et al. Revealing the doping effect of encapsulated lead halogenides on single-walled carbon nanotubes, Appled Physics A, V. 125, article number 320, 2019, Springer Nature [145]) and RbI (**c**) (reprinted with permission from [168], copyright 2019 Wiley-VCH Verlag GmbH & Co. KGaA, Weinheim) acquired at 1.96 eV-laser. The positions of the components are denoted.

An analysis of Raman modes allows the doping effects of the filled SWCNTs to be investigated in detail (Figure 5) [145,168]. In the RBM-band of the pristine SWCNTs (Figure 5a), there are peaks at 154, 171, 185, and 196 cm^−1^. In the G-band, there are three components, G^−^_LO_, G^+^_TO_, and G^+^_LO_, positioned at 1544, 1566, and 1592 cm^−1^ [168,190,193]. 

In the RBM-band of the PbCl_2_-filled SWCNTs (Figure 5b), there are peaks at 165 and 173 cm^−1^, and relative intensities are changed from 1:1.83 to 1.63:1. In the G-band, there are three peaks at 1554, 1575, and 1602. They are upshifted by 10, 9, and 10 cm^−1^ as compared to the pristine nanotubes. The relative intensity of the metallic component decreases from 0.40 to 0.06 because of the transition into a semiconducting state [159]. This is similar for other metal halogenides [122,123,124,129,135,136,137,138,140,141,142,143,144,149,150,151,158,161,165,179] and metal chalcogenides [176,180]. 

In the RBM-band of the RbI-filled SWCNTs (Figure 5c), peak positions are shifted by 5–10 cm^−1^. However, the intensities are slightly altered due to resonance conditions. In the G-band, peaks are upshifted by 9, 5, and 1 cm^−1^ as compared to the pristine nanotubes. This was consistent with the n-doping of SWCNTs observed using the encapsulated RbI. 

Modifications were also found to depend on the p-doping level of nanotubes with CuCl [129]. The RBM-peaks were upshifted at low doping levels, and they were completely suppressed at high doping levels. In the G-band, there is a gradual upshift when the doping level increases. 

#### 4.3.3. Near Edge X-ray Absorption Fine Structure Spectroscopy

Near edge X-ray absorption fine structure spectroscopy (NEXAFS) is a synchrotron-based technique for the investigation of the electronic properties of filled SWCNTs. It provides information about hybridization in filled SWCNTs and the formation of new chemical bonds between the introduced substances and SWCNTs. It also reveals modifications in the band structure of SWCNTs upon filling; moreover, it can differ between chemical bonds for different substances and elements. 

NEXAFS allows the formation of chemical bonds between nanotubes and encapsulated substances to be investigated. In the literature, the C 1s NEXAFS spectra of SWCNTs filled with iron halogenides [138], nickel halogenides [135], zinc halogenides [137], cadmium halogenides [141], silver halogenides [123], copper halogenides [124], ErCl_3_ [121], and HgCl_2_ [140] were reported. 

**Figure 6:**
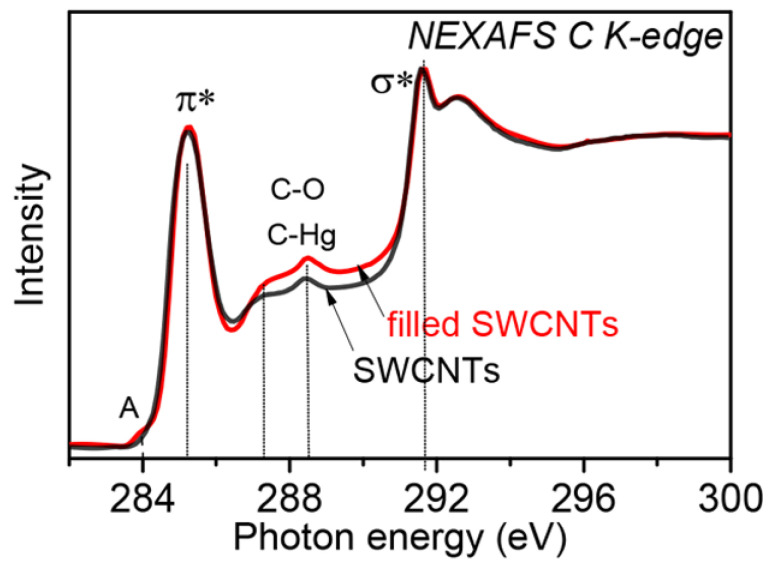
The C 1s NEXAFS spectra of SWCNTs and HgCl_2_-filled SWCNTs. The label A marks a new feature. Reprinted with permission from Fedoseeva Y.V. et al. Single-walled carbon nanotube reactor for redox transformation of mercury dichloride, ACS Nano. 2017. V.11. N.9. P.8643-8649. Copyright 2017 American Chemical Society [140].

Figure 6 compares the C 1s NEXAFS spectra of SWCNTs and HgCl_2_-filled SWCNTs [140]. There is also the π*-resonance at ~285 eV and the π*-resonance at ~292 eV. There is, however, an additional pre-edge peak in filled SWCNTs (see label A in Figure 6) before the π*-resonance at ~284.0 eV. This is due to the hybridization of the π-orbitals of SWCNTs with embedded mercury chloride. 

There is an emergence of similar additional peaks for other metal halogenides [121,123,124,135,137,138,141], because chemical bonds are formed between SWCNTs and embedded substances. 

#### 4.3.4. Photoemission Spectroscopy

The X-ray photoelectron spectroscopy (XPS) and ultraviolet photoelectron spectroscopy (UPS) allow the electronic properties of filled SWCNTs to be investigated. In XPS, the modifications in the spectra of regions are observed. They are characteristic for different fillers. This includes shifts of peaks and alterations of spectral shapes. In UPS, the changes in the valence band spectra are revealed. These changes highlight the modifications of the band structure. 

Using photoemission spectroscopy (XPS and UPS) SWCNTs filled with Ag [109,110,179], Cu [109,115], and Eu [120] were investigated, and more reports are expected.

**Figure 7:**
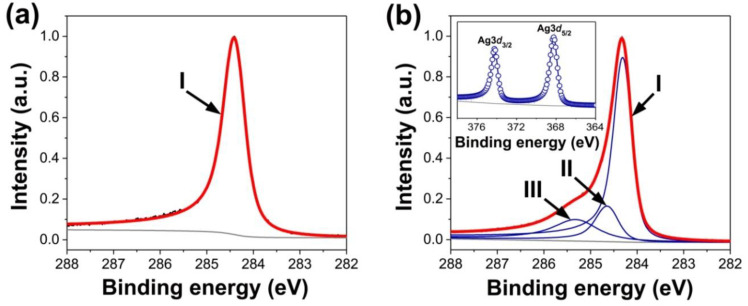
The C 1s XPS spectra of SWCNTs (**a**) and Ag-filled SWCNTs (**b**). The inset in (**b**) shows the Ag 3d XPS spectrum. Reproduced from M. V. Kharlamova et al. Donor doping of single-walled carbon nanotubes by filling of channels with silver, Journal of Experimental and Theoretical Physics, V. 115, № 3, p. 485-491, 2012, Springer Nature [110].

In Figure 7, in the C 1s XPS spectrum, the first component is unchanged and is assigned to carbon in the unfilled nanotubes, whereas the other two components belong to the silver-filled SWCNTs, whose chemical composition is confirmed by the Ag 3d XPS spectrum (see inset in Figure 7b) [110]. The second component appears upshifted by +0.33 eV due to the raised Fermi level. The third component is not interpreted [110]. The same effects are observed for copper-filled SWCNTs [109,115]. 

The C 1s peak of the Eu-filled SWCNTs was upshifted by 0.1 eV due to the incorporated europium walls as well as the further modification of the electronic structure [120]. The UPS data of the Eu-filled SWCNTs demonstrated a uniform upshift of peaks. These peaks are the consequence of the equal Fermi level upshift. These changes in the spectra are a clear signature of metallic atomic wires [120]. 

XPS quantifies the Fermi level shift and reveals the direction of the charge transfer for SWCNTs filled with manganese halogenides [136,143], iron halogenides [138], CoBr_2_ [158], nickel halogenides [135], zinc halogenides [137], silver halogenides [123], lead halogenides [145], cadmium halogenides [141], ZnCl_2_, CdCl_2_, TbCl_3_ [142], copper halogenides [124], RbI [168], RbAg_4_I_5_ [175], TmCl_3_ [179], PrCl_3_ [150], and HgCl_2_ [140] that were reported. The authors observed the shift of components. These modifications were attributed to the alteration of the electronic properties. 

The authors of Refs. [123,135,136,137,138,141,142,143,158] used the same interpretation of the components of the spectra as in the above-described case. They revealed the shift of the second component to lower binding energies, i.e., p-doping. The authors of Refs. [124,150] fitted the C 1s spectra with components of metallic and semiconducting SWCNTs. They showed larger p-doping for the metallic carbon nanotubes. 

The determination of the Fermi level shift was conducted using the secondary electrons’ (SE) cutoff for the copper halogenide-filled SWCNTs [124]. This was reported to be −0.2, −0.6, and −0.65 eV for CuI, CuBr, and CuCl, accordingly. In [137], the SE cutoff spectra of SWCNTs and ZnBr_2_-filled SWCNTs allowed the Fermi level shift of −0.3 eV to be evaluated. 

Additional valence band (VB) spectra measurements on copper halogenide- [124] and zinc bromide-filled [137] nanotubes were in line with the direct measurements of the work function in the SE cutoff spectra. Regarding the π -peaks in the VB spectra of SWCNTs, the copper halogenide-filled nanotubes originate from the photoemission from the π-band of SWCNTs, and the σ-peaks originate from the photoemission from the σ-band of SWCNTs [124]. The -peaks of filled SWCNTs are shifted to higher kinetic energies by 0.2–0.7 eV. This testifies to p-doping. The comparable effect was derived for ZnBr_2_ [137] and ErCl_3_ [121].

## 5. Quantification of Charge Transfer in SWCNTs Filled with Inorganic Compounds

**Figure 8:**
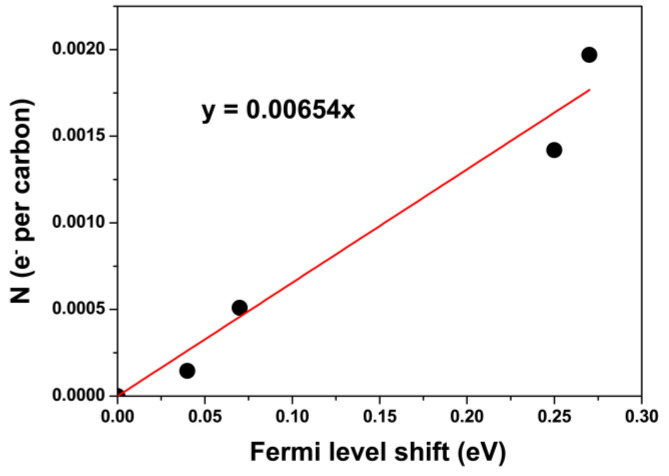
The calibration plot for potassium. The data are from Ref. [195] are plotted.

For the applications of filled nanotubes, one should quantify the charge transfer. In [132], the calculation was performed using the photoemission data. Alkali metals are very reactive, and the charge transfer is assumed to be practically one electron per K^+^ ion; thus, Fermi level shifts can be linked to charge transfer densities [195]. Using the calibration plot (Figure 8), the number of transferred electrons for AgCl-filled SWCNTs was calculated to be 0.0024 *e*^−^ per carbon. The charge transfer density along the SWCNT amounted to 0.0406 *e^−^*/Å [132].

The crucial properties of fillers that influence the doping type and doping level of SWCNTs are the work function of inorganic compounds and elementary substances. There are three doping effect-defining parameters: metallicity type, diameter of SWCNTs, and filling ratio. The introduction of metals leads to a high-yield filling of the nanotubes. It was shown that metals result in n-doping, and the Fermi level shift amounted to ~+0.3 eV. 

The encapsulation of inorganic compounds inside SWCNTs leads to a homogenous filling of SWCNTs with large filling ratios. The investigation of the filled SWCNTs, using OAS, Raman spectroscopy, XPS, UPS, and NEXAFS, proved that halogenides of 3*d*-metals (MX_2_, where M = manganese, iron, cobalt, nickel, copper, zinc, X = chlorine, bromine, iodine), 4*d*-metals (MX_2_, where M = silver, cadmium, X = chlorine, bromine, iodine), 5*d*-metals (MX_2_, where M = mercury, X = chlorine), 4*f*-metals (MX_3_, where M = praseodymium, terbium, erbium, thulium, X = chlorine, bromine, iodine), 5*p*-metals (MX_2_, where M = tin, X = fluorine), 6*p*-metals (MX_2_, where M = lead, X = chlorine, bromine, iodine), ternary halides (RbAg_4_I_5_), and gallium chalcogenides (GaX, X = selenium, tellurium) cause p-doping with a Fermi level shift of ~0.1–0.4 eV. For halogenides of 3*d*-, 4*d*- and 4*f*-metals, the Fermi level shift is the highest for chlorides and the smallest for iodides. Moreover, the hybridization of the π-orbitals of SWCNTs with introduced salts was revealed. The nanotubes of different diameters with arc-discharge and chemical vapor deposition synthesis methods have different doping levels. The introduced RbI leads to the Fermi level shift of ~+0.2 eV. 

## 6. Conclusions

This review has demonstrated how filling leads to a precise control over their electronic properties and how it has widened the scope of possible applications, raising great interest in science and technology as a result. The ability to tailor the electronic properties according to the specific requirements of individual applications renders these fascinating materials viable in the advancement of cutting-edge and fundamental applied research.

Transparency, conductivity, and mechanical robustness are key challenges in the application of nanoelectronics. While the diameter is only 1 to 2 nm, the contact length and channel length can be reduced further. Filled SWCNTs can have better contacts, requiring shorter contact lengths and changes in mechanical stiffness. Thermoelectric applications based on filled SWCNTs have to aim at a higher conversion efficiency and a decreased thermal conductivity. Electrochemical energy storage in a filled SWCNT facilitates effective charge transfer throughout the bulk of composite material. In applications in catalysis, it is desirable to improve the lifetime of the catalytic particles. It is crucial to maximize the filling ratio and purity of the filled SWCNTs. For gas sensing, one aims to achieve sensitivity and selectivity. It is also required to determine the appropriate filling for spintronic applications. For magnetic recording, the filling might offer a way to shift the paramagnetic limit. Bioimaging applications of filled SWCNT will greatly benefit from higher spatial resolution as well as from the imaging of deeper layers of tissue. Reduced cytotoxicity and better biodegradability constitute the desired improved biocompability of filled SWCNTs. The targeting systems have to be specifically developed for the different use cases. The therapeutic options may be further expanded in combined therapies, where two or more therapeutics are co-delivered on a single platform. Filled SWCNTs are also a potential electrode material for solar cells. 

## Figures and Tables

**Table 1:** Synthesis temperatures of nanocomposites X@SWCNT using the melt filling method.

Filled Substance	T_filling_, °C	Reference	Filled Substance	T_filling_, °C	Reference
manganese (II) chloride	750	[136,143]	cadmium (II) bromide	669	[141]
manganese (II) bromide	798	[136,143]	cadmium (II) iodide	488	[141]
iron (II) chloride	774	[138]	lead (II) chloride	601	[145]
iron (II) bromide	784	[138]	lead (II) bromide	471	[145]
iron (II) iodide	687	[138]	lead (II) iodide	502	[145]
cobalt (II) bromide	778	[158]	terbium (III) chloride	688	[142,149,151]
nickel (II) chloride	1101	[135]	terbium (III) bromide	927	[151]
nickel (II) bromide	1063	[135,159]	terbium (III) iodide	1057	[151]
copper (I) chloride	530	[124]	praseodymium(III) chloride	886	[149,150]
copper (I) bromide	600	[124]	erbium (III) chloride	900	[121]
copper (I) iodide	705	[124]	thulium (III) chloride	924	[149,179]
zinc (II) chloride	400	[137,142]	luthetium (III) chloride	940	[152]
zinc (II) bromide	494	[137]	luthetium (III) bromide	1050	[152]
zinc (II) iodide	546	[137]	luthetium (III) iodide	1100	[152]
rubidium (I) iodide	756	[168,175]	mercury (II) chloride	290	[140]
rubidium-silver iodide	756	[175]	silver (I) chloride	555	[123]
tin (II) fluoride	300	[122]	silver (I) bromide	530	[123]
cadmium (II) chloride	668	[141,142,144]	silver (I) iodide	660	[123]

## Data Availability

Data are available on request to first author (Marianna V. Kharlamova) of every reviewed paper.

## References

[1] Jeon I., Xiang R., Shawky A., Matsuo Y., Maruyama S. (2019). Single-Walled Carbon Nanotubes in Emerging Solar Cells: Synthesis and Electrode Applications. Adv. Energy Mater..

[2] Ferguson V., Silva S.R.P., Zhang W. (2019). Carbon Materials in Perovskite Solar Cells: Prospects and Future Challenges. Energy Environ. Mater..

[3] Fukumaru T., Fujigaya T., Nakashima N. (2015). Development of n-type cobaltocene-encapsulated carbon nanotubes with remarkable thermoelectric property. Sci. Rep..

[4] Lee J.W., Jeon I., Lin H.S., Seo S., Han T.H., Anisimov A., Kauppinen E.I., Matsuo Y., Maruyama S., Yang Y. (2019). Vapor-Assisted Ex-Situ Doping of Carbon Nanotube toward Efficient and Stable Perovskite Solar Cells. Nano Lett..

[5] Jordan J.W., Lowe G.A., McSweeney R.L., Stoppiello C.T., Lodge R.W., Skowron S.T., Biskupek J., Rance G.A., Kaiser U., Walsh D.A. (2019). Host-Guest Hybrid Redox Materials Self-Assembled from Polyoxometalates and Single-Walled Carbon Nanotubes. Adv. Mater..

[6] Martincic M., Tobias G. (2015). Filled carbon nanotubes in biomedical imaging and drug delivery. Expert Opin. Drug Deliv..

[7] Liu B.L., Wu F.Q., Gui H., Zheng M., Zhou C.W. (2017). Chirality-Controlled Synthesis and Applications of Single-Wall Carbon Nanotubes. ACS Nano.

[8] Bati A.S.R., Yu L.P., Batmunkh M., Shapter J.G. (2019). Recent Advances in Applications of Sorted Single-Walled Carbon Nanotubes. Adv. Funct. Mater..

[9] Moore K.E., Tune D.D., Flavel B.S. (2015). Double-Walled Carbon Nanotube Processing. Adv. Mater..

[10] Kharlamova M.V. (2016). Advances in tailoring the electronic properties of single-walled carbon nanotubes. Prog. Mater. Sci..

[11] Tkachenko N.V. (2006). Optical Spectroscopy: Methods and Instrumentations.

[12] Kharlamova M.V., Kramberger C., Sauer M., Yanagi K., Pichler T. (2015). Comprehensive spectroscopic characterization of high purity metallicity-sorted single-walled carbon nanotubes. Phys. Status Solidi B Basic Solid State Phys..

[13] Ayala P., Miyata Y., De Blauwe K., Shiozawa H., Feng Y., Yanagi K., Kramberger C., Silva S.R.P., Follath R., Kataura H. (2009). Disentanglement of the electronic properties of metallicity-selected single-walled carbon nanotubes. Phys. Rev. B.

[14] Kuzmany H. (2009). Solid-State Spectroscopy: An Introduction.

[15] Atalla R.H., Agarwal U.P., Bond J.L. (1992). Raman Spectroscopy.

[16] Smekal A. (1923). The quantum theory of dispersion. Naturwissenschaften.

[17] Raman C.V., Krishnan K.S. (1928). The optical analog of the Compton eect. Nature.

[18] Lamdsberg G., Mandelstam L. (1928). A novel efect of light scattering in crystals. Naturwissenschaften.

[19] Maiman T.H. (1960). Stimulated optical radiation in ruby. Nature.

[20] Porto S.P.S., Wood D.L. (1962). Ruby optical maser as a raman source. J. Opt. Soc. Am..

[21] Smith E., Dent G. (2005). Modern Raman Spectroscopy: A Practical Approach.

[22] Kharlamova M.V., Mochalin V.N., Lukatskaya M.R., Niu J., Presser V., Mikhalovsky S., Gogotsi Y. (2013). Adsorption of proteins in channels of carbon nanotubes: Effect of surface chemistry. Mater. Express.

[23] Burdanova M.G., Tsapenko A.P., Kharlamova M.V., Kauppinen E.I., Gorshunov B.P., Kono J., Lloyd-Hughes J. (2021). A review of the terahertz conductivity and photoconductivity of carbon nanotubes and heteronanotubes. Adv. Opt. Mater..

[24] Fouquet M., Telg H., Maultzsch J., Wu Y., Chandra B., Hone J., Heinz T.F., Thomsen C. (2009). Longitudinal Optical Phonons in Metallic and Semiconducting Carbon Nanotubes. Phys. Rev. Lett..

[25] Kharlamova M.V., Burdanova M.G., Paukov M.I., Kramberger C. (2022). Synthesis, Sorting, and Applications of Single-Chirality Single-Walled Carbon Nanotubes. Materials.

[26] Das A., Sood A.K. (2009). Renormalization of the phonon spectrum in semiconducting single-walled carbon nanotubes studied by Raman spectroscopy. Phys. Rev. B.

[27] Grimm S., Schiessl S.P., Zakharko Y., Rother M., Brohmann M., Zaumseil J. (2017). Doping-dependent G-mode shifts of small diameter semiconducting single-walled carbon nanotubes. Carbon.

[28] Tsang J.C., Freitag M., Perebeinos V., Liu J., Avouris P. (2007). Doping and phonon renormalization in carbon nanotubes. Nat. Nanotechnol..

[29] Das A., Sood A.K., Govindaraj A., Saitta A.M., Lazzeri M., Mauri F., Rao C.N.R. (2007). Doping in carbon nanotubes probed by Raman and transport measurements. Phys. Rev. Lett..

[30] Kalbac M., Farhat H., Kavan L., Kong J., Dresselhaus M.S. (2008). Competition between the Spring Force Constant and the Phonon Energy Renormalization in Electrochemically Doped Semiconducting Single-Walled Carbon Nanotubes. Nano Lett..

[31] Zhang L., Liao V., Yu Z.H. (2010). Raman spectroelectrochemistry of a single-wall carbon nanotube bundle. Carbon.

[32] Kharlamova M.V., Paukov M., Burdanova M.G. (2022). Nanotube Functionalization: Investigation, Methods and Demonstrated Applications. Materials.

[33] Lazzeri M., Piscanec S., Mauri F., Ferrari A.C., Robertson J. (2006). Phonon linewidths and electron-phonon coupling in graphite and nanotubes. Phys. Rev. B.

[34] Caudal N., Saitta A.M., Lazzeri M., Mauri F. (2007). Kohn anomalies and nonadiabaticity in doped carbon nanotubes. Phys. Rev. B.

[35] Nguyen K.T., Gaur A., Shim M. (2007). Fano lineshape and phonon softening in single isolated metallic carbon nanotubes. Phys. Rev. Lett..

[36] Farhat H., Son H., Samsonidze G.G., Reich S., Dresselhaus M.S., Kong J. (2007). Phonon softening in individual metallic carbon nanotubes due to the Kohn anomaly. Phys. Rev. Lett..

[37] Watts J.F., Wolstenholme J. (2003). An Introduction to Surface Analysis by XPS and AES.

[38] Leckrey R., O’Connor D.J., Sexton B.A., Smart R.S.C. (1992). Ultraviolet Photoelectron Spectroscopy of Solids. Surface Analysis Methods in Materials Science.

[39] Hertz H. (1887). Uber einen einuss des ultravioletten lichtes auf die electrische entladung. Ann. Phys..

[40] Einstein A. (1905). Uber einen die erzeugung und verwandlung des lichtes betreenden heuristischen gesichtspunkt. Ann. Phys..

[41] Innes P.D. (1907). On the velocity of the cathode particles emitted by various metals under the inuence of Röntgen rays, and its bearing on the theory of atomic disintegration. Proc. R. Soc. Lond. Ser. A.

[42] Briggs D., Grant G.T. (2003). Perspectives on XPS and AES. Surface Analysis by Auger and X-ray Photoelectron Spectroscopy.

[43] Steinhardt R.G., Serfass E.J. (1953). Surface analysis with X-ray photoelectron spectrometer. Anal. Chem..

[44] Nordling C., Sokolowski E., Siegbahn K. (1957). Precision method for obtaining absolute values of atomic binding energies. Rhys. Rev..

[45] Siegbahn K., Nordling C., Fahlman A., Nordberg L., Hamrin K., Hedman J., Johansson J., Bergmark T., Karlsson S.E., Lindgren I. (1967). Electron Spectroscopy for Chemical Analysis. Atomic, Molecular and Solid State Structure Studies by Means of Electron Spectroscopy.

[46] Briggs D. (2003). XPS: Basic Principles, Spectral Features and Qualitative Analysis. Surface Analysis by Auger and X-ray Photoelectron Spectroscopy.

[47] Vilesov F.I., Kurbatov B.L., Terenin A.N. (1961). Electron Distribution Over Energies In Photoionization Of Aromatic Amines in Gaseous Phase. Sov. Phys. Dokl..

[48] Spicer W.E., Beglund C.N. (1964). d-Band of copper. Phys. Rev. Lett..

[49] Berglund C.N., Spicer W.E. (1964). Photoemission studies of copper and silver. Phys. Rev..

[50] Turner D.W., Baker C., Baker A.D., Brundle C.R. (1970). Molecular Photoelectron Spectroscopy.

[51] Turner D.W., Al-Joboury M.I. (1962). Determination of ionization potentials by photoelectron energy measurement. J. Chem. Phys..

[52] Heber M., Grunert W. (2001). Application of ultraviolet photoelectron spectroscopy (UPS) in the surface characterisation of polycrystalline oxide catalysts. I. Methodics and analytical potential. Top. Catal..

[53] Park Y., Choong V., Gao Y. (2021). Work function of indium tin oxide transparent conductor measured by photoelectron spectroscopy. Appl. Phys. Lett..

[54] Kramberger C., Rauf H., Shiozawa H., Knupfer M., Buchner B., Pichler T., Batchelor D., Kataura H. (2007). Unraveling van Hove singularities in x-ray absorption response of single-wall carbon nanotubes. Phys. Rev. B.

[55] Su W.S., Leung T.C., Chan C.T. (2007). Work function of single-walled and multiwalled carbon nanotubes: First-principles study. Phys. Rev. B.

[56] Doniach S., Sunjic M. (1970). Many-Electron Singularity in X-ray Photoemission and X-ray Line Spectra from Metals. J. Phys. Part C Solid State Phys..

[57] Ishii H., Kataura H., Shiozawa H., Yoshioka H., Otsubo H., Takayama Y., Miyahara T., Suzuki S., Achiba Y., Nakatake M. (2003). Direct observation of Tomonaga-Luttinger-liquid state in carbon nanotubes at low temperatures. Nature.

[58] Kharlamova M.V., Kramberger C. (2021). Spectroscopy of Filled Single-Walled Carbon Nanotubes. Nanomaterials.

[59] Schnohr C.S., Ridgway M. (2015). X-ray Absorption Spectroscopy of Semiconductors.

[60] Stohr J. (1992). NEXAFS Spectroscopy.

[61] Wang M.Y., Arnadottir L., Xu Z.C.J., Feng Z.X. (2019). In Situ X-ray Absorption Spectroscopy Studies of Nanoscale Electrocatalysts. Nano-Micro Lett..

[62] Bianconi A. (1980). Surface X-ray Absorption-Spectroscopy—Surface Exafs and Surface Xanes. Appl. Surf. Sci..

[63] De Broglie M. (1913). Sur un nouveau procédé permettant d’obtenir la photographie des spectres de raies des rayons Röntgen. C. R. Acad. Sci..

[64] Fricke H. (1920). The K-Characteristic Absorption Frequencies for the Chemical Elements Magnesium to Chromium. Phys. Rev..

[65] Hertz G. (1920). Uber die Absorptionsgrenzen in der L-Serie. Z. Phys..

[66] Hanawalt J.D. (1931). The Dependence of X-ray Absorption Spectra upon Chemical and Physical State. Phys. Rev..

[67] Kronig R.d.L. (1931). Zur Theorie der Feinstruktur in den Rontgenabsorptionsspektren. Z. Phys..

[68] Kronig R.d.L. (1932). Zur Theorie der Feinstruktur in den R¡§ontgenabsorptionsspektren. II. Z. Phys..

[69] Kronig R.d.L. (1932). Zur Theorie der Feinstruktur in den R¡§ontgenabsorptionsspektren. III. Z. Phys..

[70] Petersen H. (1932). Zur Theorie der R¡§ontgenabsorption molekularer Gase. Z. Phys..

[71] Petersen H. (1933). Zur Theorie der R¡§ontgenabsorption molekularer Gase. II. Z. Phys..

[72] Petersen H. (1936). Zur Theorie der Rontgenabsorption molekularer Gase. III. Z. Phys..

[73] Cauchois Y., Mott N.F. (1949). The Interpretation of X-ray Absorption Spectra of Solids. Phil. Mag..

[74] Lytle F.W., Ferraro J.R., Ziomek J.S. (1963). X-ray Absorption Fine-Structure Investigations at Cryogenic Temperatures. Developments in Applied Spectroscopy.

[75] Sawada M., Tsutsumi K., Shiraiwa T., Obashi M. (1955). On the Fine Structures of X-ray Absorption Spectra of Amorphous Substances: The Amorphous State of the Binary System of Nickel-Sulfur. II. J. Phys. Soc. Jpn..

[76] Shiraiwa T. (1960). The Theory of the Fine Structure of the X-ray Absorption Spectrum, II. J. Phys. Soc. Jpn..

[77] Snyder T.M., Shaw C.H. (1940). The Fine Structure of the X-ray Absorption Limits of Bromine and Chlorine. Phys. Rev..

[78] Lytle F. (1966). Determination of Interatomic Distances from X-ray Absorption Fine Structure. Adv. X-ray Anal..

[79] Van Nordstand R.A. (1960). The Use of X-ray K-Absorption Edges in the Study of Catalytically Active Solids. Adv. Catal..

[80] Van Nordstrand R., Kaelble E. (1967). Handbook of X-Rays.

[81] Sayers D.E., Stern E.A., Lytle F.W. (1971). New Technique for Investigating Noncrystalline Structures: Fourier Analysis of the Extended X-ray Absorption Fine Structure. Phys. Rev. Lett..

[82] Lytle F.W., Sayers D.E., Stern E.A. (1975). Extended X-ray-Absorption Fine-Structure Technique. II. Experimental Practice and Selected Results. Phys. Rev. B.

[83] Rehr J.J., Stern E.A., Martin E.L., Davidson E.R. (1978). Extended X-ray-Absorption Fine-Structure Amplitudes, Wave-Function Relaxation and Chemical Effects. Phys. Rev. B.

[84] Sandstrom D.R., Lytle F.W. (1979). Developments in Extended X-ray Absorption Fine Structure Applied to Chemical Systems. Ann. Rev. Phys. Chem..

[85] Stern E.A. (1974). Theory of the Extended X-ray-Absorption Fine Structure. Phys. Rev. B.

[86] Stern E.A., Sayers D.E., Lytle F.W. (1975). Extended X-ray-Absorption Fine-Structure Technique. III. Determination of Physical Parameters. Phys. Rev. B.

[87] Ashley C.A., Doniach S. (1975). Theory of Extended X-ray Absorption Edge Fine Structure (EXAFS) in Crystalline Solids. Phys. Rev. B.

[88] Beni G., Platzman P.L. (1976). Temperature and Polarization Dependence of Extended X-ray Absorption Fine-Structure Spectra. Phys. Rev. B.

[89] Lee P.A., Pendry J.B. (1975). Theory of the Extended X-ray Absorption Fine Structure. Phys. Rev. B.

[90] Schaich W.L. (1973). Comment on the Theory of Extended X-ray-Absorption Fine Structure. Phys. Rev. B.

[91] Sevillano G., Meuth H., Rehr J. (1979). Extended X-ray Absorption Fine Structure Debye-Waller Factors. I. Monatomic Crystals. Phys. Rev. B.

[92] Lamberti C., van Bockhoven J.A., van Bockhoven J.A., Lamberti C. (2016). Introduction: Historical Perspective on XAS. X-ray Absorption and X-ray Emission Spectroscopy: Theory and Applications.

[93] Yano J., Yachandra V.K. (2009). X-ray absorption spectroscopy. Photosynth. Res..

[94] Smith B.W., Luzzi D.E. (2000). Formation mechanism of fullerene peapods and coaxial tubes: A path to large scale synthesis. Chem. Phys. Lett..

[95] Yudasaka M., Ajima K., Suenaga K., Ichihashi T., Hashimoto A., Iijima S. (2003). Nano-extraction and nano-condensation for C-60 incorporation into single-wall carbon nanotubes in liquid phases. Chem. Phys. Lett..

[96] Zhang Y., Iijima S., Shi Z., Gu Z. (1999). Defects in arc-discharge-produced single-walled carbon nanotubes. Philos. Mag. Lett..

[97] Simon F., Kramberger C., Pfeiffer R., Kuzmany H., Zolyomi V., Kurti J., Singer P.M., Alloul H. (2005). Isotope engineering of carbon nanotube systems. Phys. Rev. Lett..

[98] Kharlamova M.V., Kramberger C., Sato Y., Saito T., Suenaga K., Pichler T., Shiozawa H. (2018). Chiral vector and metal catalyst-dependent growth kinetics of single-walled carbon nanotube. Carbon.

[99] Kharlamova M.V., Kramberger C., Saito T., Sato T., Suenaga K., Pichler T., Shizawa H. (2017). Chirality-dependent growth of single-walled carbon nanotubes as revealed insie nano-test tubes. Nanoscale.

[100] Wang Z.Y., Shi Z.J., Gu Z.N. (2010). Synthesis of single-walled carbon nanotube/metal nanoparticle hybrid materials from potassium-filled nanotubes. Carbon.

[101] Kiang C.H., Choi J.S., Tran T.T., Bacher A.D. (1999). Molecular nanowires of 1 nm diameter from capillary filling of single-walled carbon nanotubes. J. Phys. Chem. B.

[102] Borowiak-Palen E., Mendoza E., Bachmatiuk A., Rummeli M.H., Gemming T., Nogues J., Skumryev V., Kalenczuk R.J., Pichler T., Silva S.R.P. (2006). Iron filled single-wall carbon nanotubes—A novel ferromagnetic medium. Chem. Phys. Lett..

[103] Borowiak-Palen E., Bachmatiuk A., Rummeli M.H., Gemming T., Pichler T., Kalenczuk R.J. (2006). Iron filled singlewalled carbon nanotubes—Synthesis and characteristic properties. Phys. Status Solidi B Basic Solid State Phys..

[104] Cui T.T., Pan X.L., Dong J.H., Miao S., Miao D.Y., Bao X.H. (2018). A versatile method for the encapsulation of various non-precious metal nanoparticles inside single-walled carbon nanotubes. Nano Res..

[105] Li Y.F., Kaneko T., Ogawa T., Takahashi M., Hatakeyama R. (2008). Novel properties of single-walled carbon nanotubes with encapsulated magnetic atoms. Jpn. J. Appl. Phys..

[106] Domanov O., Weschke E., Saito T., Peterlik H., Pichler T., Eisterer M., Shiozawa H. (2019). Exchange coupling in a frustrated trimetric molecular magnet reversed by a 1D nano-confinement. Nanoscale.

[107] Sloan J., Hammer J., Zwiefka-Sibley M., Green M.L.H. (1998). The opening and filling of single walled carbon nanotubes (SWTs). Chem. Commun..

[108] Govindaraj A., Satishkumar B.C., Nath M., Rao C.N.R. (2000). Metal nanowires and intercalated metal layers in single-walled carbon nanotube bundles. Chem. Mater..

[109] Kharlamova M.V., Niu J.J. (2012). Comparison of metallic silver and copper doping effects on single-walled carbon nanotubes. Appl. Phys. A.

[110] Kharlamova M.V., Niu J.J. (2012). Donor doping of single-walled carbon nanotubes by filling of channels with silver. J. Exp. Theor. Phys..

[111] Borowiak-Palen E., Ruemmeli M.H., Gemming T., Pichler T., Kalenczuk R.J., Silva S.R.P. (2006). Silver filled single-wall carbon nanotubes—Synthesis, structural and electronic properties. Nanotechnology.

[112] Corio P., Santos A.P., Santos P.S., Temperini M.L.A., Brar V.W., Pimenta M.A., Dresselhaus M.S. (2004). Characterization of single wall carbon nanotubes filled with silver and with chromium compounds. Chem. Phys. Lett..

[113] Sloan J., Wright D.M., Woo H.G., Bailey S., Brown G., York A.P.E., Coleman K.S., Hutchison J.L., Green M.L.H. (1999). Capillarity and silver nanowire formation observed in single walled carbon nanotubes. Chem. Commun..

[114] Zhang Z.L., Li B., Shi Z.J., Gu Z.N., Xue Z.Q., Peng L.M. (2000). Filling of single-walled carbon nanotubes with silver. J. Mater. Res..

[115] Kharlamova M.V., Niu J.J. (2012). New method of the directional modification of the electronic structure of single-walled carbon nanotubes by filling channels with metallic copper from a liquid phase. JETP Lett..

[116] Chamberlain T.W., Zoberbier T., Biskupek J., Botos A., Kaiser U., Khlobystov A.N. (2012). Formation of uncapped nanometre-sized metal particles by decomposition of metal carbonyls in carbon nanotubes. Chem. Sci..

[117] Costa P.M.F.J., Sloan J., Rutherford T., Green M.L.H. (2005). Encapsulation of RexOy clusters within single-walled carbon nanotubes and their in tubulo reduction and sintering to Re metal. Chem. Mater..

[118] Zoberbier T., Chamberlain T.W., Biskupek J., Kuganathan N., Eyhusen S., Bichoutskaia E., Kaiser U., Khlobystov A.N. (2012). Interactions and Reactions of Transition Metal Clusters with the Interior of Single-Walled Carbon Nanotubes Imaged at the Atomic Scale. J. Am. Chem. Soc..

[119] Kitaura R., Nakanishi R., Saito T., Yoshikawa H., Awaga K., Shinohara H. (2009). High-Yield Synthesis of Ultrathin Metal Nanowires in Carbon Nanotubes. Aew. Chem. Int. Ed..

[120] Nakanishi R., Kitaura R., Ayala P., Shiozawa H., De Blauwe K., Hoffmann P., Choi D., Miyata Y., Pichler T., Shinohara H. (2012). Electronic structure of Eu atomic wires encapsulated inside single-wall carbon nanotubes. Phys. Rev. B.

[121] Ayala P., Kitaura R., Nakanishi R., Shiozawa H., Ogawa D., Hoffmann P., Shinohara H., Pichler T. (2011). Templating rare-earth hybridization via ultrahigh vacuum annealing of ErCl3 nanowires inside carbon nanotubes. Phys. Rev. B.

[122] Zakalyukin R.M., Mavrin B.N., Dem’yanets L.N., Kiselev N.A. (2008). Synthesis and characterization of single-walled carbon nanotubes filled with the superionic material SnF2. Carbon.

[123] Eliseev A.A., Yashina L.V., Brzhezinskaya M.M., Chernysheva M.V., Kharlamova M.V., Verbitsky N.I., Lukashin A.V., Kiselev N.A., Kumskov A.S., Zakalyuhin R.M. (2010). Structure and electronic properties of AgX (X = Cl, Br, I)-intercalated single-walled carbon nanotubes. Carbon.

[124] Eliseev A.A., Yashina L.V., Verbitskiy N.I., Brzhezinskaya M.M., Kharlamova M.V., Chernysheva M.V., Lukashin A.V., Kiselev N.A., Kumskov A.S., Freitag B. (2012). Interaction between single walled carbon nanotube and 1D crystal in CuX@SWCNT (X = Cl, Br, I) nanostructures. Carbon.

[125] Flahaut E., Sloan J., Coleman K., Green M. (2001). Synthesis of 1D P-block halide crystals within single walled carbon nanotubes. AIP Conf. Proc..

[126] Monthioux M., Flahaut E., Cleuziou J.P. (2006). Hybrid carbon nanotubes: Strategy, progress, and perspectives. J. Mater. Res..

[127] Sloan J., Kirkland A.I., Hutchison J.L., Green M.L.H. (2002). Integral atomic layer architectures of 1D crystals inserted into single walled carbon nanotubes. Chem. Commun..

[128] Eremina V.A., Fedotov P.V., Obraztsova E.D. (2016). Copper chloride functionalization of semiconducting and metallic fractions of single-walled carbon nanotubes. J. Nanophotonics.

[129] Fedotov P.V., Tonkikh A.A., Obraztsova E.A., Nasibulin A.G., Kauppinen E.I., Chuvilin A.L., Obraztsova E.D. (2014). Optical properties of single-walled carbon nanotubes filled with CuCl by gas-phase technique. Phys. Status Solidi B Basic Solid State Phys..

[130] Fedotov P.V., Eremina V.A., Tonkikh A.A., Chernov A.I., Obraztsova E.D. (2016). Enhanced optical transparency of films formed from sorted metallic or semiconducting single-walled carbon nanotubes filled with CuCl. Phys. Status Solidi B Basic Solid State Phys..

[131] Kharlamova M.V., Kramberger C., Mittelberger A., Yanagi K., Pichler T., Eder D. (2018). Silver Chloride Encapsulation-Induced Modifications of Raman Modes of Metallicity-Sorted Semiconducting Single-Walled Carbon Nanotubes. J. Spectrosc..

[132] Kharlamova M.V., Kramberger C., Domanov O., Mittelberger A., Yanagi K., Pichler T., Eder D. (2018). Fermi level engineering of metallicity-sorted metallic single-walled carbon nanotubes by encapsulation of few-atom-thick crystals of silver chloride. J. Mater. Sci..

[133] Kharlamova M.V., Kramberger C., Domanov O., Mittelberger A., Saito T., Yanagi K., Pichler T., Eder D. (2018). Comparison of Doping Levels of Single-Walled Carbon Nanotubes Synthesized by Arc-Discharge and Chemical Vapor Deposition Methods by Encapsulated Silver Chloride. Phys. Status Solidi B Basic Solid State Phys..

[134] Burdanova M.G., Kharlamova M.V., Kramberger C., Nikitin M.P. (2021). Applications of pristine and functionalized carbon nanotubes, graphene, and graphene nanoribbons in biomedicine. Nanomaterials.

[135] Kharlamova M.V., Yashina L.V., Eliseev A.A., Volykhov A.A., Neudachina V.S., Brzhezinskaya M.M., Zyubina T.S., Lukashin A.V., Tretyakov Y.D. (2012). Single-walled carbon nanotubes filled with nickel halogenides: Atomic structure and doping effect. Phys. Status Solidi B Basic Solid State Phys..

[136] Kharlamova M.V., Eliseev A.A., Yashina L.V., Lukashin A.V., Tretyakov Y.D. (2012). Synthesis of Nanocomposites on Basis of Single-Walled Carbon Nanotubes Intercalated by Manganese Halogenides.

[137] Kharlamova M.V., Yashina L.V., Volykhov A.A., Niu J.J., Neudachina V.S., Brzhezinskaya M.M., Zyubina T.S., Belogorokhov A.I., Eliseev A.A. (2012). Acceptor doping of single-walled carbon nanotubes by encapsulation of zinc halogenides. Eur. Phys. J. B.

[138] Kharlamova M.V., Brzhezinskaya M., Vinogradov A., Suzdalev I., Maksimov Y.V., Imshennik V., Novichikhin S.V., Krestinin A.V., Yashina L.V., Lukashin A.V. (2009). The forming and properties of one-dimensional FeHaI 2 (HaI=Cl, Br, I) nanocrystals in channels of single-walled carbon nanotubes. Russ. Nanotechnol..

[139] Sloan J., Friedrichs S., Flahaut E., Brown G., Bailey S.R., Coleman K.S., Xu C., Green M.L.H., Hutchison J.L., Kirkland A.I. (2001). The characterisation of sub-nanometer scale structures within single walled carbon nanotubes. AIP Conf. Proc..

[140] Fedoseeva Y.V., Orekhov A.S., Chekhova G.N., Koroteev V.O., Kanygin M.A., Seovskiy B.V., Chuvilin A., Pontiroli D., Ricco M., Bulusheva L.G. (2017). Single-Walled Carbon Nanotube Reactor for Redox Transformation of Mercury Dichloride. ACS Nano.

[141] Kharlamova M.V., Yashina L.V., Lukashin A.V. (2013). Charge transfer in single-walled carbon nanotubes filled with cadmium halogenides. J. Mater. Sci..

[142] Kharlamova M.V. (2013). Comparison of influence of incorporated 3d-, 4d- and 4f- metal chlorides on electronic properties of single-walled carbon nanotubes. Appl. Phys. A.

[143] Kharlamova M.V. (2016). Electronic properties of single-walled carbon nanotubes filled with manganese halogenides. Appl. Phys. A.

[144] Kharlamova M.V., Kramberger C., Pichler T. (2016). Semiconducting response in single-walled carbon nanotubes filled with cadmium chloride. Phys. Status Solidi B Basic Solid State Phys..

[145] Kharlamova M.V., Kramberger C., Rudatis P., Pichler T., Eder D. (2019). Revealing the doping effect of encapsulated lead halogenides on single-walled carbon nanotubes. Appl. Phys. A.

[146] Kitaura R., Ogawa D., Kobayashi K., Saito T., Ohshima S., Nakamura T., Yoshikawa H., Awaga K., Shinohara H. (2008). High Yield Synthesis and Characterization of the Structural and Magnetic Properties of Crystalline ErCl3 Nanowires in Single-Walled Carbon Nanotube Templates. Nano Res..

[147] Satishkumar B.C., Taubert A., Luzzi D.E. (2003). Filling single-wall carbon nanotubes with d- and f-metal chloride and metal nanowires. J. Nanosci. Nanotech..

[148] Xu C.G., Sloan J., Brown G., Bailey S., Williams V.C., Friedrichs S., Coleman K.S., Flahaut E., Hutchison J.L., Dunin-Borkowski R.E. (2000). 1D lanthanide halide crystals inserted into single-walled carbon nanotubes. Chem. Commun..

[149] Kharlamova M.V. (2015). Rare-earth metal halogenide encapsulation-induced modifications in Raman spectra of single-walled carbon nanotubes. Appl. Phys. A.

[150] Kharlamova M.V., Volykhov A.A., Yashina L.V., Egorov A.V., Lukashin A.V. (2015). Experimental and theoretical studies on the electronic properties of praseodymium chloride-filled single-walled carbon nanotubes. J. Mater. Sci..

[151] Kharlamova M.V., Kramberger C., Mittelberger A. (2017). Raman spectroscopy study of the doping effect of the encapsulated terbium halogenides on single-walled carbon nanotubes. Appl. Phys. A.

[152] Santidrian A., Kierkowicz M., Pach E., Darvasiova D., Ballesteros B., Tobias G., Kalbac M. (2020). Charge transfer in steam purified arc discharge single walled carbon nanotubes filled with lutetium halides. Phys. Chem. Chem. Phys..

[153] Brown G., Bailey S.R., Sloan J., Xu C.G., Friedrichs S., Flahaut E., Coleman K.S., Hutchison J.L., Dunin-Borkowski R.E., Green M.L.H. (2001). Electron beam induced in situ clusterisation of 1D ZrCl4 chains within single-walled carbon nanotubes. Chem. Commun..

[154] Brown G., Bailey S.R., Novotny M., Carter R., Flahaut E., Coleman K.S., Hutchison J.L., Green M.L.H., Sloan J. (2003). High yield incorporation and washing properties of halides incorporated into single walled carbon nanotubes. Appl. Phys. A.

[155] Kirkland A.I., Meyer M.R., Sloan J., Hutchison J.L. (2005). Structure determination of atomically controlled crystal architectures grown within single wall carbon nanotubes. Microsc. Microanal..

[156] Sloan J., Friedrichs S., Meyer R.R., Kirkland A.I., Hutchison J.L., Green M.L.H. (2002). Structural changes induced in nanocrystals of binary compounds confined within single walled carbon nanotubes: A brief review. Inorg. Chim. Acta.

[157] Sloan J., Kirkland A.I., Hutchison J.L., Green M.L.H. (2003). Aspects of crystal growth within carbon nanotubes. C. R. Phys..

[158] Kharlamova M.V., Eliseev A.A., Yashina L.V., Petukhov D.I., Liu C.P., Wang C.Y., Semenenko D.A., Belogorokhov A.I. (2010). Study of the electronic structure of single-walled carbon nanotubes filled with cobalt bromide. JETP Lett..

[159] Kharlamova M.V. (2015). Raman Spectroscopy Study of the Doping Effect of the Encapsulated Iron, Cobalt, and Nickel Bromides on Single-Walled Carbon Nanotubes. J. Spectrosc..

[160] Bendall J.S., Ilie A., Welland M.E., Sloan J., Green M.L.H. (2006). Thermal stability and reactivity of metal halide filled single-walled carbon nanotubes. J. Phys. Chem. B.

[161] Chernysheva M.V., Eliseev A.A., Lukashin A.V., Tretyakov Y.D., Savilov S.V., Kiselev N.A., Zhigalina O.M., Kumskov A.S., Krestinin A.V., Hutchison J.L. (2007). Filling of single-walled carbon nanotubes by Cul nanocrystals via capillary technique. Phys. E.

[162] Hutchison J.L., Sloan J., Kirkland A.I., Green M.L.H., Green M.L.H. (2004). Growing and characterizing one-dimensional crystals within single-walled carbon nanotubes. J. Electron Microsc..

[163] Kiselev N.A., Zakalyukin R.M., Zhigalina O.M., Grobert N., Kumskov A.S., Grigoriev Y.V., Chernysheva M.V., Eliseev A.A., Krestinin A.V., Tretyakov Y.D. (2008). The structure of 1D CuI crystals inside SWNTs. J. Microsc..

[164] Kiselev N.A., Kumskov A.S., Zakalyukin R.M., Vasiliev A.L., Chernisheva M.V., Eliseev A.A., Krestinin A.V., Freitag B., Hutchison J.L. (2012). The structure of nanocomposite 1D cationic conductor crystal@SWNT. J. Microsc..

[165] Kumskov A.S., Zhigalina V.G., Chuvilin A.L., Verbitskiy N.I., Ryabenko A.G., Zaytsev D.D., Eliseev A.A., Kiselev N.A. (2012). The structure of 1D and 3D CuI nanocrystals grown within 1.5-2.5 nm single wall carbon nanotubes obtained by catalyzed chemical vapor deposition. Carbon.

[166] Meyer R.R., Sloan J., Dunin-Borkowski R.E., Kirkland A.I., Novotny M.C., Bailey S.R., Hutchison J.L., Green M.L.H. (2000). Discrete atom imaging of one-dimensional crystals formed within single-walled carbon nanotubes. Science.

[167] Sloan J., Novotny M.C., Bailey S.R., Brown G., Xu C., Williams V.C., Friedrichs S., Flahaut E., Callender R.L., York A.P.E. (2000). Two layer 4:4 co-ordinated KI crystals grown within single walled carbon nanotubes. Chem. Phys. Lett..

[168] Kharlamova M.V., Kramberger C., Rudatis P., Yanagi K., Eder D. (2019). Characterization of the Electronic Properties of Single-Walled Carbon Nanotubes Filled with an Electron Donor-Rubidium Iodide: Multifrequency Raman and X-ray Photoelectron Spectroscopy Studies. Phys. Status Solidi B Basic Solid State Phys..

[169] Flahaut E., Sloan J., Friedrichs S., Kirkland A.I., Coleman K.S., Williams V.C., Hanson N., Hutchison J.L., Green M.L.H. (2006). Crystallization of 2H and 4H PbI2 in carbon nanotubes of varying diameters and morphologies. Chem. Mater..

[170] Philp E., Sloan J., Kirkland A.I., Meyer R.R., Friedrichs S., Hutchison J.L., Green M.L.H. (2003). An encapsulated helical one-dimensional cobalt iodide nanostructure. Nat. Mater..

[171] Sloan J., Grosvenor S.J., Friedrichs S., Kirkland A.I., Hutchison J.L., Green M.L.H. (2002). A one-dimensional BaI2 chain with five- and six-coordination, formed within a single-walled carbon nanotube. Aew. Chem. Int. Ed..

[172] Friedrichs S., Falke U., Green M.L.H. (2005). Phase separation of Lal(3) inside single-walled carbon nanotubes. Chemphyschem.

[173] Friedrichs S., Kirkland A.I., Meyer R.R., Sloan J., Green M.L.H. (2005). LaI2@(18,3)SWNT: The unprecedented structure of a LaI2 “Crystal,” encapsulated within a single-walled carbon nanotube. Microsc. Microanal..

[174] Sloan J., Terrones M., Nufer S., Friedrichs S., Bailey S.R., Woo H.G., Ruhle M., Hutchison J.L., Green M.L.H. (2002). Metastable one-dimensional AgCl1-xIx solid-solution wurzite “tunnel” crystals formed within single-walled carbon nanotubes. J. Am. Chem. Soc..

[175] Falaleev N.S., Kumskov A.S., Zhigalina V.G., Verbitskiy I.I., Vasiliev A.L., Makarova A.A., Vyalikh D.V., Kiselev N.A., Eliseev A.A. (2017). Capsulate structure effect on SWNTs doping in RbxAg1-xI@SWNT composites. Crystengcomm.

[176] Kharlamova M.V. (2014). Comparative analysis of electronic properties of tin, gallium, and bismuth chalcogenide-filled single-walled carbon nanotubes. J. Mater. Sci..

[177] Eliseev A.A., Chernysheva M.V., Verbitskii N.I., Kiseleva E.A., Lukashin A.V., Tretyakov Y.D., Kiselev N.A., Zhigalina O.M., Zakalyukin R.M., Vasiliev A.L. (2009). Chemical Reactions within Single-Walled Carbon Nanotube Channels. Chem. Mater..

[178] Wang Z.Y., Li H., Liu Z., Shi Z.J., Lu J., Suenaga K., Joung S.K., Okazaki T., Gu Z.N., Zhou J. (2010). Mixed Low-Dimensional Nanomaterial: 2D Ultranarrow MoS2 Inorganic Nanoribbons Encapsulated in Quasi-1D Carbon Nanotubes. J. Am. Chem. Soc..

[179] Kharlamova M.V., Yashina L.V., Lukashin A.V. (2013). Comparison of modification of electronic properties of single-walled carbon nanotubes filled with metal halogenide, chalcogenide, and pure metal. Appl. Phys. A.

[180] Kharlamova M.V. (2013). Novel approach to tailoring the electronic properties of single-walled carbon nanotubes by the encapsulation of high-melting gallium selenide using a single-step process. JETP Lett..

[181] Carter R., Sloan J., Kirkland A.I., Meyer R.R., Lindan P.J.D., Lin G., Green M.L.H., Vlandas A., Hutchison J.L., Harding J. (2006). Correlation of structural and electronic properties in a new low-dimensional form of mercury telluride. Phys. Rev. Lett..

[182] Sloan J., Carter R., Meyer R.R., Vlandas A., Kirkland A.I., Lindan P.J.D., Lin G., Harding J., Hutchison J.L. (2006). Structural correlation of band-gap modifications induced in mercury telluride by dimensional constraint in single walled carbon nanotubes. Phys. Status Solidi B Basic Solid State Phys..

[183] Yashina L.V., Eliseev A.A., Kharlamova M.V., Volykhov A.A., Egorov A.V., Savilov S.V., Lukashin A.V., Puttner R., Belogorokhov A.I. (2011). Growth and Characterization of One-Dimensional SnTe Crystals within the Single-Walled Carbon Nanotube Channels. J. Phys. Chem. C.

[184] Kumskov A.S., Eliseev A.A., Freitag B., Kiselev N.A. (2012). HRTEM of 1DSnTe@SWNT nanocomposite located on thin layers of graphite. J. Microsc..

[185] Li L.J., Lin T.W., Doig J., Mortimer I.B., Wiltshire J.G., Taylor R.A., Sloan J., Green M.L.H., Nicholas R.J. (2006). Crystal-encapsulation-induced band-structure change in single-walled carbon nanotubes: Photoluminescence and Raman spectra. Phys. Rev. B.

[186] Li L.J., Lin T.W., Doig J., Mortimer I.B., Wiltshire J.G., Taylor R.A., Sloan J., Green M.L.H., Nicholas R.J. (2007). Band structure changes in carbon nanotubes caused by MnTe2 crystal encapsulation. AIP Conf. Proc..

[187] Kanda N., Nakanishi Y., Liu D., Liu Z., Inoue T., Miyata Y., Tomanek D., Shinohara H. (2020). Efficient growth and characterization of one-dimensional transition metal tellurides inside carbon nanotubes. Nanoscale.

[188] Nagata M., Shukla S., Nakanishi Y., Liu Z., Lin Y.C., Shiga T., Nakamura Y., Koyama T., Kishida H., Inoue T. (2019). Isolation of Single-Wired Transition-Metal Monochalcogenides by Carbon Nanotubes. Nano Lett..

[189] Araujo P.T., Maciel I.O., Pesce P.B.C., Pimenta M.A., Doorn S.K., Qian H., Hartschuh A., Steiner M., Grigorian L., Hata K. (2008). Nature of the constant factor in the relation between radial breathing mode frequency and tube diameter for single-wall carbon nanotubes. Phys. Rev. B.

[190] Brown S.D.M., Corio P., Marucci A., Dresselhaus M.S., Pimenta M.A., Kneipp K. (2000). Anti-Stokes Raman spectra of single-walled carbon nanotubes. Phys. Rev. B.

[191] Piscanec S., Lazzeri M., Robertson J., Ferrari A.C., Mauri F. (2007). Optical phonons in carbon nanotubes: Kohn anomalies, Peierls distortions, and dynamic effects. Phys. Rev. B.

[192] Dresselhaus M.S., Dresselhaus G., Saito R., Jorio A. (2005). Raman spectroscopy of carbon nanotubes. Phys. Rep..

[193] Dresselhaus M.S., Dresselhaus G., Jorio A., Souza A.G., Saito R. (2002). Raman spectroscopy on isolated single wall carbon nanotubes. Carbon.

[194] Kataura H., Kumazawa Y., Maniwa Y., Umezu I., Suzuki S., Ohtsuka Y., Achiba Y. (1999). Optical properties of single-wall carbon nanotubes. Synthet. Met..

[195] Rauf H., Pichler T., Knupfer M., Fink J., Kataura H. (2004). Transition from a Tomonaga-Luttinger liquid to a Fermi liquid in potassium-intercalated bundles of single-wall carbon nanotubes. Phys. Rev. Lett..

